# School Refusal and Absenteeism: Perception of Teacher Behaviors, Psychological Basic Needs, and Academic Achievement

**DOI:** 10.3389/fpsyg.2019.01471

**Published:** 2019-06-27

**Authors:** Pina Filippello, Caterina Buzzai, Sebastiano Costa, Luana Sorrenti

**Affiliations:** ^1^Department of Clinical and Experimental Medicine, University of Messina, Messina, Italy; ^2^Department of Cognitive Sciences, Psychological, Educational and Cultural Studies, University of Messina, Messina, Italy; ^3^Department of Psychology, University of Campania “Luigi Vanvitelli”, Caserta, Italy

**Keywords:** school refusal, self-determination theory, psychological basic needs, absenteeism, teacher perceived psychological control, perceived teacher support

## Abstract

School refusal (SR) is a complex problem that may be caused by different risk factors such as individual and contextual factors (Kearney, 2007; Maynard et al., 2018; Heyne et al., 2019). These mechanisms can be described in the context of self-determination theory (SDT). For these reasons, the purpose of the present study is investigate the relationship between teacher perceived psychological control and support, psychological basic needs, SR behavior, and academic achievement, on adolescent sample. It is hypothesized that teacher perceived psychological control and autonomy support play a role on need frustration and need satisfaction; in turn, need satisfaction could reduce while need frustration could promote SR behavior and number of absences. Finally, SR behavior and number of absences could reduce academic achievement. 263 students (196 females, 67 males) with an average age of 16.14 (SD = 1.35; range 13–20 years). SEM analyses with observed variables have shown that the final model fit well the data, χ^2^(8) = 16.34, *p* = 0.04, CFI = 0.96, SRMR = 0.04, RMSEA (90% CI) = 0.06 (0.01; 0.10), showing the following significant path: need satisfaction was positively predicted by perceived teacher support and negatively predicted by teacher perceived psychological control; need frustration was positively predicted by teacher perceived psychological control; number of absences was negatively predicted by need satisfaction; SR was positively predicted by need frustration; school achievement was negatively predicted by SR and number of absences. These results have several implications for the school context and the deepening of the construct of SR and absenteeism.

## Introduction

In accordance with the Functional Model of School Refusal Behavior (Kearney, 2008; Kearney and Spear, 2014), *school refusal* (SR) can be defined as a school attendance problem that manifests in various ways, such as: not attending school for a long time; not staying in class all the time; arriving late to school; and students attending school only because they are forced to by their parents (Kearney and Albano, 2010). SR differs from other school attendance problems (e.g., truancy, school withdrawal, or school exclusion) due to some of its specific characteristics: (a) students show reluctance to attend school and resistive behavior when parents try to get them to attend; (b) students stay at home when not attending school, and the parents know about it; (c) school causes these students emotional distress, such as somatic complaints and anxiety; and (d) students do not exhibit antisocial behavior (Heyne et al., 2019; Ingul et al., 2019).

The Functional Model of School Refusal Behavior describes four main reasons why children develop SR (Kearney, 2008; Kearney and Spear, 2014): (a) to avoid general school-related distress caused by known or unknown factors (i.e., school is where they experience feelings of rejection or shame); (b) to escape from adverse social situations and/or the school evaluation system (i.e., unstructured circumstances, group work, writing on the board); (c) to draw the attention of parents (i.e., children have non-compliance, escape, or physical symptoms that occur at home to avoid separation); and (d) to obtain gratification out of school. In this last case, the refusal relates specifically to the possibility of continuing pleasant experiences perceived as more rewarding than attending school, such as watching television or hanging out with friends.

In the study of SR, one must take into account individual and contextual factors, and self-determination theory (SDT) (Deci and Ryan, 2000; Vansteenkiste et al., 2010; Ryan and Deci, 2017) proves useful for analyzing the interaction of these factors. In accordance with SDT, the individual’s effective functioning depends on the satisfaction of three fundamental psychological needs – autonomy, relatedness, and competence – and SDT contends that the interpersonal context plays a fundamental role in satisfying these needs. The interpersonal context can be defined as either controlling or supportive depending on whether it contributes to the satisfaction or, conversely, to the frustration of psychological needs (Vansteenkiste et al., 2010; Ryan and Deci, 2017; Costa et al., 2019). For example, support from significant adults (parents and teachers) is essential for the satisfaction of basic psychological needs; conversely, harsh educational practices may thwart these needs (Costa et al., 2016; Ryan and Deci, 2017; Filippello et al., in press).

An undoubtedly dysfunctional educational practice is psychological control, which involves intrusive practices that rely on the manipulation of youths’ psychological and emotional states (Soenens and Vansteenkiste, 2010). Psychological control is considered a destructive form of control, rendering young people vulnerable to ill-being (Barber, 1996; Rogers et al., 2003).

Despite literature demonstrating that all types of psychological control (whether by parents or teachers) are positively associated with school maladjustment and underachievement (Filippello et al., 2015, 2018a), teacher control seems to play a more significant role in the development of feelings of incompetence, helplessness, and frustration in attempting school tasks (Filippello et al., 2014, 2017; Sorrenti et al., 2018).

Psychologically controlling teachers adopt covert behaviors (e.g., the induction of guilt, limiting overt verbal expression, hindering the critical and independent views of the students, exhibiting disapproval, or ignoring students who do not reach or do not behave according to their standards) to manipulate their students and ensure compliance with their directives (Soenens et al., 2012; Filippello et al., 2019). In accordance with SDT, teachers’ psychological control can hinder the satisfaction of psychological needs, encouraging an external locus of motivation rather than intrinsic motivation (Reeve et al., 2004; Reeve and Jang, 2006; Filippello et al., 2019). Psychological teaching control produces, in fact, a learning context characterized by control, obligation, and coercion, eliciting in the students shame, guilt, anxiety (Soenens et al., 2012), insecurity, and fear of failure (Ryan et al., 1992; Filippello et al., 2017). Several studies have found that the negative emotions arising from perceived controlling teaching related, in turn, to low school engagement, less use of learning strategies, and lower grades (Assor et al., 2005; Reeve, 2009; Soenens et al., 2012; Filippello et al., 2017).

On the contrary, teachers who create a supportive learning environment pay attention to their students’ points of view and needs, encourage conversation, and make use of praise as informational feedback, encouragement, and hints on ways to improve. These methods favor higher levels of interest, intrinsic motivation, the formation of an internal locus of control, self-efficacy, and commitment among the students, because they support the self-realization of students’ goals (Ryan et al., 1992; Reeve et al., 2004; Reeve and Jang, 2006; Filippello et al., 2019).

The literature shows that supportive student–teacher relationships can play a protective role, thus alleviating the onset of stress in young people (Murberg and Bru, 2009). Conversely, problematic student–teacher relationships could promote stress, depression (Fiorilli et al., 2019) and negative emotions toward school, resulting in feelings of frustration and helplessness (Sorrenti et al., 2015a,b). Therefore, these negative relationships and related consequences could also represent a risk factor for SR (Ingul et al., 2019). However, although several studies have identified a relationship between SR and a lack of teacher support, fear of the teacher (Havik et al., 2015), and conflict with teachers (Baker and Bishop, 2015), the link between SR and the perceived exertion of psychological control by teachers has not been investigated sufficiently. The literature shows that teachers’ psychological controlling behavior usually rewards students who satisfy their high expectations and achieve excellent results (Filippello et al., 2017). Therefore, such behavior can induce a sense of guilt and shame in students who fail to achieve high standards, a situation that could frustrate the three basic psychological needs and create a sense of helplessness in the students. Consequently, school could become a source of frustration, and lead students to avoidance behavior and to the onset of SR. Therefore, it would be suitable to implement studies to verify whether the teacher perceived psychological control, frustrating the basic psychological needs, can favor SR. Indeed, it has been observed that teacher perceived psychological control, through the mediating role of other variables (e.g., helplessness) is a predictor of academic underachievement (Filippello et al., 2019).

Most students with SR do not attend school, and the increase in absences has an effect on learning and academic achievement (Heyne et al., 2019; Ingul et al., 2019). Many studies have shown the link between SR and poor academic performance (Barry et al., 2010; Yahaya et al., 2010; Thornton et al., 2013). Furthermore, students with SR are more frequently exposed to the risk of presenting external behavioral problems or emotional maladjustments (Maynard et al., 2012; Nelemans et al., 2014). The early identification of individuals with SR is very important for the prevention of the negative consequences of SR behaviors, such as dropping out of school (Gonzálvez et al., 2018).

### The Present Study

The studies mentioned above demonstrate that teachers’ psychological control could contribute to the development of SR. However, although the literature has shown that the lack of support from teachers is a risk factor in SR development (Havik et al., 2015), there is a dearth of research on the mediating role of the student’s perception of teacher psychological control in SR, and its relationship with academic achievement.

For these reasons, the purpose of the present study is to investigate the mediating role of need satisfaction and need frustration at school in the relationship between student’s perception of teacher control and teacher support, SR behavior (as global score and four functional conditions: Avoidance, Escape, Attention-seeking, and Gratification), number of absences, and the impact on academic achievement in an adolescent sample. It is hypothesized that student’s perception of teacher control and teacher support play a role in need frustration and need satisfaction; need satisfaction could decrease SR behavior (both the global score and the single conditions), while need frustration could promote it, and increase absences. Furthermore, SR behavior and the number of absences could reduce academic achievement. Finally, it was hypothesized the mediation role of need satisfaction and need frustration in the association between the student’s perception of teacher control/support and SR behavior/number of absence and, also the mediation role of need satisfaction/frustration and SR behavior/number of absence in the association between the student’s perception of teacher control/support and academic achievement.

## Materials and Methods

### Participants

The sample consisted of 263 students – 196 females (74.5%) and 67 males (25.5%) – with an average age of 16.14 (SD = 1.35; range 13–20 years). Participants were selected from a high school in Messina with various orientations of study (linguistic, scientific, classical, artistic, social sciences), Sicily (Italy), through a random sampling procedure. 95.4% of the students were Italian, and all were Italian speaking. Furthermore, 15.2% of the students had low socioeconomic status (SES) (one or both parents held a lower secondary education diploma), 43.7% had medium SES (one or both parents held a high school diploma), and 41.1% had high SES (one or both parents held a university degree).

### Instruments

In this study, some of the scales employed have been adapted in Italian. According to the recommendations of the International Test Commission (Hambleton, 2001), the Italian versions of the *Teacher as Social Context Questionnaire, Psychological Control Teaching Scale–Student Report* and *The Basic Psychological Need Satisfaction and Frustration Scale for the school context* were adapted using the back-translation method. The questionnaires were adapted from English to Italian by three independent translators, expert in the SDT. Each translator translated the measures from English to Italian and successively they discussed all the discrepancies identified until finding a satisfactory solution. This procedure from Italian to English proved to be identical in content with the three questionnaires original versions.

The *Demographic Questionnaire* was administered to collect basic demographic information from the participants, including age, gender, national origin, educational level/academic class, and SES.

An adapted version of the *Teacher as Social Context Questionnaire* (TASCQ; Belmont et al., 1988) was used to assess students’ perceived need of teacher support. We used the five positively worded items from the TASCQ on autonomy support (e.g., *“My teacher gives me a lot of choices about how I do my schoolwork”*). Participants responded on a 5-point Likert-type scale, ranging from 1 (strongly disagree) to 5 (strongly agree). Scale scores were computed as the means of the items. The reliability and validity of this scale have been documented in several countries (Aelterman et al., 2014; Haerens et al., 2015).

The *Psychological Control Teaching Scale–Student Report* (PCTS–SR; Soenens et al., 2012) was used to evaluate the student’s perception of teacher psychological control. The scale consists of seven items (e.g., *“My teacher clearly shows that I have hurt their feelings when I have failed to live up to their expectations”*) and the participants responded on a 5-point Likert-type scale, ranging from 1 (strongly disagree) to 5 (strongly agree). Scale scores were computed as the means of the items. Soenens et al. (2012) provided evidence for the validity of this scale, and the reliability has been documented in different countries, including Italy (Filippello et al., 2017, 2019).

An adapted version of *The Basic Psychological Need Satisfaction and Frustration Scale* (BPNSFS; Costa et al., 2018) for the school context was used in this study. It contains 24 items assessing the student’s perception of satisfaction (12 items; e.g., *“I feel a sense of choice and freedom in the things I undertake at school”*) and frustration (12 items; e.g., *“At school, I feel forced to do many things I wouldn’t choose to do*”) relating to psychological needs in the school context. Participants responded on a 5-point Likert scale ranging from 1 (completely disagree) to 5 (completely agree). For this study, the total average of the items was computed to obtain two scores – Need Satisfaction at School and Need Frustration at School. The reliability and validity of BPNSFS have been documented in different countries (Chen et al., 2015; Cordeiro et al., 2016; Liga et al., 2018).

The *number of absences* was based on the total number of absences by the students during the school year in question (from September to April).

The *School Refusal Behavior Scale-Revised – SRAS* (Kearney, 2007), specifically the Italian version by Rigante and Patrizi (2007), was used to evaluate a student’s risk of SR behavior. This consists of 24 items rated on a 7-point Likert-type scale ranging from 0 (never) to 6 (always). The scale measures four functional dimensions: avoidance of negative affectivity-provoking stimuli or situations related to a school setting (e.g., *“How often do you have trouble going to school because you are afraid of something in the school building, for example teacher, school bus, etc.?”*); escape from aversive, social, or evaluative situations (e.g., *“Do you have trouble speaking with the other kids at school?”*); attention-seeking behavior (e.g., *“Do you often do things to upset or annoy your family?”*); and positive tangible reinforcement/gratification (e.g., *“Do you ever skip school because it’s more fun to be out of school?”*). For this study, scores for each sub-scale were computed as the means of items and the SR total score average was computed. The reliability and validity of this scale have been documented in different countries (Rigante and Patrizi, 2007; Kearney and Albano, 2010; Sorrenti et al., 2016; Filippello et al., 2018b, 2019).

#### Academic Achievement

The data on academic achievement were provided by the students based on the average scores earned on written tests and oral questions across all subjects during the school year in question.

### Procedure

This study was carried out in accordance with the recommendations of the Ethical Code of the Italian Association of Psychology (AIP), with written informed consent from all subjects. All subjects gave written informed consent in accordance with the Declaration of Helsinki (2013). The protocol was approved by the Ethics Committee of the Centre for Research and Psychological Intervention (CERIP) of the University of Messina (protocol number: 30465). Approval from the school was requested and received to conduct the study. Furthermore, all of the students were given informed consent to take part in the research. Written informed consent was obtained from the parents of all the participants in this study. Data collection took place in April, 7 months after the start of the school year. Participants completed all of the questionnaires in a single session lasting 20–30 min. Academic achievement and number of absences data were provided by students using online access to the school register. Privacy and the anonymity of their answers were guaranteed.

### Data Analysis

RStudio with the lavaan package was used to carry out the path analysis with maximum likelihood estimation and 5000 resample of bootstrapped estimates. Several indexes of fit were examined: the Chi-square (χ^2^) value; the Comparative Fit Index (CFI); Standardized Root Mean Square Residual (SRMR) and the Root Mean Square Error of Approximation (RMSEA) with its 90% confidence interval (CI) (for a description of these indices, see Hair et al., 1998). Cut-off for a good model fit is achieved when the CFI values is >0.90, the SRMR and the RMSEA are <0.08 (Kline, 2015). IBM SPSS was used to conduct descriptive statistics, Cronbach’s alpha, and correlations for all variables in the study.

## Results

### Descriptive Statistics, Reliability, and Correlation

Table 1 shows means, standard deviation, skewness, kurtosis, Cronbach’s alpha values for all measures considerate in this study. The descriptive analysis showed that all scales had good scores for symmetry and kurtosis, and the reliability of the measures was adequate. Mardia’s coefficients for multivariate skew (b1p = 4.74) and kurtosis (b2p = 72.44) revealed that the data departed significantly from multivariate normality and to account for multivariate non-normality of the data, the maximum likelihood estimation with bootstrapped resamples approach was used. Correlations showed that the avoidance was positively correlated with need frustration, teacher perceived psychological control and number of absences, while it was negatively related with need satisfaction and perceived teacher support; escape was positively related with need frustration, teacher perceived psychological control and number of absences, while it was negatively correlated with need satisfaction and academic achievement; attention-seeking was positively related with need frustration and teacher perceived psychological control, while it was negatively correlated with academic achievement; gratification wasn’t related with any of the variables considered.

**Table 1:** Descriptive statistics and correlation among variables.

	Min	Max	M	SD	Skew	Kurt	1	2	3	4	5	6	7	8	9	10
1. Perceived teacher support	1.00	5.00	2.74	0.82	0.03	–0.28	α = 0.79									
2. Teacher perceived psychological control	1.00	5.00	2.37	0.77	0.58	0.11	–0.36**	α = 0.78								
3. Need satisfaction at school	1.83	4.67	3.64	0.57	–0.62	0.50	0.26**	–0.23**	α = 0.81							
4. Need frustration at school	2.71	3.29	2.88	0.12	0.88	0.52	–0.22**	0.35**	–0.44**	α = 0.82						
5. Avoidance	0.17	5.50	2.65	1.24	0.08	–0.67	–0.24**	0.41**	–0.29**	0.45**	α = 0.76					
6. Escape	0.00	6.00	1.01	1.00	1.76	4.30	–0.02	0.22**	–0.32**	0.47**	0.41**	α = 0.72				
7. Attention-seeking	0.00	5.50	1.82	1.26	0.68	–0.25	–0.02	0.17**	–0.10	0.24**	0.50**	0.46**	α = 0.81			
8. Gratification	0.33	5.33	3.01	1.05	–0.18	–0.44	–0.04	0.10	0.02	0.00	0.11	0.02	0.08	α = 0.60		
9. School refusal	0.67	4.83	2.12	0.77	0.70	0.45	–0.13*	0.34**	–0.26**	0.43**	0.78**	0.69**	0.79**	0.42**	α = 0.82	
10. Number of absences	0.00	34.00	11.08	5.84	0.72	0.69	–0.07	0.11	–0.21**	0.17**	0.19**	0.14*	0.09	0.01	0.16**	–
11. Academic achievement	5.00	9.50	7.35	0.81	–0.07	0.73	0.05	–0.02	0.14*	–0.13*	–0.12	–0.14*	–0.17**	–0.04	–0.18**	–0.20**

*N = 263; α, Cronbach’s Alpha; M, mean; SD, standard deviation; Skew, skewness; Kurt, kurtosis; ^∗^*p* < 0.05; ^∗∗^*p* < 0.01.*

Furthermore, correlational analysis showed that the SR was positively related with need frustration, teacher perceived psychological control, and number of absences, while it was negatively correlated with need satisfaction, perceived teacher support, and academic achievement.

### Path Analyses

To investigate the mediating role of need satisfaction and need frustration at school in the relationship between teacher perceived psychological control and support, SR behavior (as global score and for the four functional conditions: Avoidance, Escape, Attention-seeking, and Gratification), number of absences, and the impact on academic achievement, two path analyses were employed. In the first path analyses the global score of the SR behavior was used, while in the second path analyses the four functional conditions: Avoidance, Escape, Attention-seeking, and Gratification were used as conceptualization of the SR behavior. In model 1 was tested a model using the following paths: Need Satisfaction at School and Need Frustration at School predicted by Perceived Teacher Support and Teacher Perceived Psychological Control; Number of Absences and SR predicted by Need Satisfaction at School and Need Frustration at School; School Grades predicted by Number of Absences and SR. Furthermore, in the hypothesized model, the following couples of variables were allowed to correlate with each other: Perceived Teacher Support and Teacher Perceived Psychological Control; Need Satisfaction at School and Need Frustration at School; and Number of Absences and SR.

**FIGURE 1:**
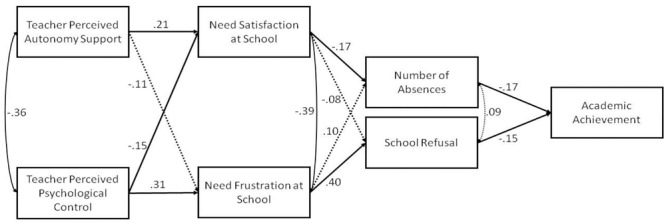
Path diagram depicting the relationships between study variables.

The results from the hypothesized model (Figure 1) showed excellent fit indices, χ^2^(8) = 16.34, *p* = 0.04, CFI = 0.96, SRMR = 0.04, RMSEA (90% CI) = 0.06 (0.01; 0.10), and indicated that need satisfaction was positively predicted by perceived teacher support (*b* = 0.15, 95% CIs [0.05; 0.24], β = 0.21, *p* < 0.01) and negatively predicted by teacher perceived psychological control (*b* = -0.11, 95% CIs [-0.22; -0.01], β = -0.15, *p* < 0.05); need frustration was positively predicted by teacher perceived psychological control (*b* = 0.05, 95% CIs [0.03; 0.07], β = 0.31, *p* < 0.01); number of absences was negatively predicted by need satisfaction (*b* = -1.76, 95% CIs [-3.21; -0.21], β = -0.17, *p* < 0.05); SR was positively predicted by need frustration (*b* = 2.56, 95% CIs [1.67; 3.45], β = 0.40, *p* < 0.01); academic achievement was negatively predicted by SR (*b* = 0.16, 95% CIs [-0.29; -0.03], β = -0.15, *p* < 0.05) and number of absences (*b* = -0.02, 95% CIs [-0.04; -0.01], β = -0.17, *p* < 0.05). Notably, an examination of the indirect effects showed: an indirect positive effect from teacher perceived psychological control to academic achievement via the mediation effect of need frustration at school and SR (*b* = 2.45, 95% CIs [1.57; 3.32], β = 0.56, *p* < 0.01); an indirect negative effect from teacher perceived psychological control to academic achievement via the mediation effect of need satisfaction at school and number of absences (*b* = -1.89, 95% CIs [-3.32; -0.29], β = -0.50, *p* < 0.05); and an indirect negative effect from perceived teacher support to academic achievement via the mediation effect of need satisfaction at school and number of absences (*b* = -1.63, 95% CIs [-3.03; -0.05], β = -0.13, *p* < 0.05).

In model 2 was tested a model using the following paths: Need Satisfaction at School and Need Frustration at School predicted by Perceived Teacher Support and Teacher Perceived Psychological Control; Number of Absences and Avoidance, Escape, Attention-seeking, and Gratification predicted by Need Satisfaction at School and Need Frustration at School; Academic Achievement predicted by Number of Absences and SR. Furthermore, in the hypothesized model, the following couples of variables were allowed to correlate with each other: Perceived Teacher Support and Teacher Perceived Psychological Control; Need Satisfaction at School and Need Frustration at School; and Number of Absences and Avoidance, Escape, Attention-seeking, and Gratification.

The results from the hypothesized model showed excellent fit indices, χ^2^(14) = 39.32, *p* < 0.001, CFI = 0.94, SRMR = 0.05, RMSEA (90% CI) = 0.08 (0.05; 0.01), and indicated that need satisfaction was positively predicted by perceived teacher support (*b* = 0.15, 95% CIs [0.05; 0.24], β = 0.21, *p* < 0.01) and negatively predicted by teacher perceived psychological control (*b* = -0.11, 95% CIs [-0.22; -0.01], β = -0.15, *p* < 0.05); need frustration was positively predicted by teacher perceived psychological control (*b* = 0.05, 95% CIs [0.03; 0.07], β = 0.31, *p* < 0.01); avoidance was positively predicted by need frustration (*b* = 4.17, 95% CIs [2.93; 5.37], β = 0.40, *p* < 0.001); escape was positively predicted by need frustration (*b* = 3.49, 95% CIs [2.27; 4.77], β = 0.41, *p* < 0.001); attention-seeking was positively predicted by need frustration (*b* = 2.55, 95% CIs [1.03; 4.08], β = 0.24, *p* < 0.01); number of absences was negatively predicted by need satisfaction (*b* = -1.76, 95% CIs [-3.22; -0.20], β = -0.17, *p* < 0.05); academic achievement was negatively predicted by number of absences (*b* = -0.03, 95% CIs [-0.04; -0.01], β = -0.18, *p* < 0.05). Furthermore, as regards the indirect effects it has been shown: an indirect negative effect from teacher perceived psychological control to academic achievement via the mediation effect of need satisfaction at school and number of absences (*b* = -1.89, 95% CIs [-3.39; -0.32], β = -0.50, *p* < 0.05); and an indirect negative effect from perceived teacher support to academic achievement via the mediation effect of need satisfaction at school and number of absences (*b* = -1.64, 95% CIs [-3.11; -0.08], β = -0.14, *p* < 0.05).

## Discussion

School refusal is a complex issue that can be determined by different individual and contextual risk factors that interact with each other; these act as predisposing, precipitating, and/or perpetuating factors (Heyne et al., 2014; Maynard et al., 2018) that could lead to a rapid decline in school adjustment and achievement (Dembo et al., 2016; Gonzálvez et al., 2017, 2018).

For these reasons, the aim of this study was to investigate the mediating role of need satisfaction and need frustration at school in the relationship between teachers perceived psychological control and support, SR behavior (as global score and the four functional conditions: avoidance, escape, attention seeking, and gratification), number of absences, and the impact on academic achievement in a sample of adolescent students. Specifically, the investigation focused on whether teacher perceived psychological control and perceived teachers support contributes to the satisfaction or frustration of psychological needs at school, and subsequently predicts SR behavior (as global score and the four functional conditions: avoidance, escape, attention seeking and gratification), number of absences, and ultimately academic achievement. To achieve this goal, two models were tested. In model 1, together with the variables mentioned above, SR total score was considered, while, in model 2 the four functional conditions of SR were considered.

The results of model 1 confirmed the role of need frustration at school as a mediator between SR and teacher perceived psychological control. This suggests that the teacher perceived psychological control has a significant influence on the frustration of psychological needs at school and, therefore, is associated with increased SR behavior. According to SDT (Ryan and Deci, 2017), when the teacher adopts a control behavior (e.g., induction of guilt, exhibiting disapproval, or ignoring students who do not reach their standards) the students may experience a sense of external or self-imposed control, doubt their ability, feel excluded from the school context, and experience shame, guilt, and anxiety (Soenens et al., 2012). Consequently, students can seek to avoid general school-related distress, escape from adverse social situations, or look for gratification outside the school (Kearney, 2008; Kearney and Spear, 2014). Furthermore, this study showed the mediating role of need satisfaction between teacher perceived psychological control and number of absences and academic achievement. This indicates that teachers’ manipulation of their students to ensure compliance with their directives (Soenens et al., 2012; Cheon and Reeve, 2015) hinders the satisfaction of students’ basic psychological needs at school, which in turn has a negative influence on school involvement (increasing the number of absences) and on academic achievement, consistent with previous studies (Niemiec and Ryan, 2009; Soenens et al., 2012; Cheon and Reeve, 2015). Another result to emerge from this study was the role of satisfaction at school as a mediator between number of absences and perceived teacher support. This result, again consistent with other studies (Niemiec and Ryan, 2009; Yu et al., 2015; Malu and Reddy, 2016; Molinari and Mameli, 2018), suggests that when teachers pay attention to their students’ point of view, they support their need to feel free to choose, be competent, and be connected with others, thus increasing their involvement at school and reducing the number of absences.

The results of model 2 showed the same direct effects as model 1 from teacher perceived psychological control and perceived teacher support to need frustration and need satisfaction. Furthermore, a direct effect was shown from need frustration to avoidance, escape and attention-seeking but the latter did not show any significant effect on the number of absences and academic achievement. Finally, this model did not confirm the role of need frustration at school and need satisfaction as a mediator between avoidance, escape, attention-seeking, gratification and teacher perceived psychological control and perceived teacher support. Instead, the role of satisfaction at school as a mediator between number of absences and perceived teacher support and role of need satisfaction between teacher perceived psychological control and number of absences and academic achievement were significant. Probably this could due to the fact that SR is a multidimensional process that refers to different aspects that, if taken individually, may not necessarily result in a reduction in academic achievement, but instead integrated together can adequately represent the complexity of the SR and therefore highlight this relationship.

Overall, the results of this study are consistent with SDT (Ryan and Deci, 2017) that asserts that an individual’s effective functioning depends on the satisfaction of basic psychological needs, which in turn are influenced by the interpersonal context. Moreover, this is the first study to investigate the relationship between teacher perceived psychological control and support, need satisfaction and need frustration at school, and SR behavior from the perspective of SDT. The findings provide an important contribution to the literature on SR behavior by suggesting that a school environment that cannot support students’ basic psychological needs can be a risk factor for SR development and poor academic achievement. Moreover, it has been shown that a supportive context can promote the satisfaction of basic psychological needs and have an influence on the number of absences.

This study has some limitations. First, the direction of the effects hypothesized in our model cannot be tested, due to the use of self-reports measures. Although the evaluation and interpretation of events play an important role in the functional and dysfunctional behaviors of individuals, the only use of students “self-assessment on the support of teachers” autonomy and the psychological control of teachers could be considered a limitation of the study. Indeed, student responses may have been more influenced by their interpretative bias than by the actual behavior of teachers. Therefore, future studies should include different measurement and evaluation methods to verify the correspondence between the interpretation of a behavior and actual behavior.

A further limitation is that the sample is small size and consists only of high school students, thus preventing the generalization of the results. Future research should include a sample of children from middle and elementary schools.

Despite these limitations, the results have important practical implications in the school context. It is clear that it is possible to implement teacher training aimed at modifying the intrusive practices that rely on the manipulation of youths’ psychological and emotional states (Barber, 1996; Soenens and Vansteenkiste, 2010) by advocating supportive practices that involve paying attention to students’ needs, encouraging conversation, and providing suggestions on ways to improve (Reeve et al., 2004; Reeve and Jang, 2006; Yu et al., 2015). Increasing teachers’ awareness of their style of teaching and modifying dysfunctional attitudes could have repercussions for the classroom climate, favoring a context in which students feel autonomous, competent, and connected with others. This, in turn, can reduce the emergence of dysfunctional behaviors such as SR and increase academic achievement.

Future research lines could examine whether other sources of support (e.g., parents and peers) can hinder the negative effects of intrusive teacher practices. Indeed, the literature show that a supportive context encourages autonomy and satisfies competence and relatedness needs. This increases the level of student engagement, promotes self-realization and facilitates positive functioning among adolescents within schools (Reeve and Jang, 2006).

## Data Availability

The datasets generated for this study are available on request to the corresponding author.

## Ethics Statement

This study was carried out in accordance with the recommendations of Codice Etico dell’Associazione Italiana di Psicologia (AIP), with written informed consent from all subjects. All subjects gave written informed consent in accordance with the Declaration of Helsinki. The protocol was approved by the University of Messina.

## Author Contributions

PF assisted with the manuscript preparation, study design, and study concept. CB assisted with the manuscript preparation, data analysis, and study design. SC assisted with the manuscript editing, data analysis, and interpretation of results. LS assisted with the manuscript preparation, study design, and study supervision.

## Conflict of Interest Statement

The authors declare that the research was conducted in the absence of any commercial or financial relationships that could be construed as a potential conflict of interest.
